# Cost-Effectiveness of Supersaturated Oxygen Delivery for Infarct Size Reduction in Patients With Anterior ST-Segment Elevation Myocardial Infarction

**DOI:** 10.1016/j.jscai.2026.105321

**Published:** 2026-05-07

**Authors:** Katherine Vilain, Harun Kundi, Bjorn Redfors, Emily M. Bucholz, Neel M. Butala, David J. Cohen

**Affiliations:** a University of Missouri-Kansas City’s Healthcare Institute for Innovations in Quality, Kansas City, Missouri; b Saint Luke’s Mid America Heart Institute, Kansas City, Missouri; c Cardiovascular Research Foundation, New York, New York; d Section of Cardiology, Department of Pediatrics, University of Colorado and Children's Hospital Colorado, Aurora, Colorado; e Rocky Mountain Regional VA Medical Center, Aurora, Colorado; f University of Colorado School of Medicine, Aurora, Colorado; g St. Francis Hospital & Heart Center, Roslyn, New York

**Keywords:** cost-effectiveness, decision analysis, heart failure, myocardial infarct size, ST-segment elevation myocardial infarction, supersaturated oxygen

## Abstract

**Background:**

Myocardial infarct size (IS) is a key determinant of long-term outcomes after acute myocardial infarction (MI). Although infusion of supersaturated oxygen (SSO_2_) after primary percutaneous coronary intervention (PPCI) has been shown to reduce IS among patients with acute anterior ST-segment elevation myocardial infarction (STEMI), the cost-effectiveness of this approach remains unknown.

**Methods:**

We developed a Markov model to project long-term mortality, quality of life, and costs among patients with acute anterior STEMI undergoing PPCI. Among surviving patients, we modeled the incidence of post-MI heart failure, cardiovascular death, and noncardiovascular death as a function of IS, using population-based data for calibration. Health state–specific costs were derived from Medicare data. The cost of SSO_2_ (including supplies, procedure time, and physician costs) was assumed to be $10,528. Deterministic and probabilistic sensitivity analyses were used to quantify the effects of uncertainty on the results.

**Results:**

In our base case analysis (mean age 61 years; 22% female; mean IS 25% of LV mass), treatment with SSO_2_ was projected to increase discounted life expectancy (14.21 vs 14.12 years) and quality-adjusted life expectancy (10.78 vs 10.69 quality-adjusted life-years [QALYs]) while increasing lifetime costs by $3205/patient. The incremental cost-effectiveness ratio for SSO_2_ vs standard care was $38,415/QALYs gained, which was sensitive to patient age, IS with standard care, and the impact of SSO_2_ on IS. For this population, the probability that SSO_2_ provides good economic value (at a societal willingness-to-pay threshold of $120,000/QALYs) was 73%.

**Conclusions:**

Based on our disease simulation model, SSO_2_ therapy for patients undergoing PPCI for acute anterior STEMI appears to be a cost-effective adjunct to PPCI from the perspective of the US health care system.

## Introduction

Heart failure (HF) is one of the most common reasons for hospitalization in older patients, and leads to increased mortality, impaired health status and quality of life (QOL), and increased health care resource utilization.^1^ In the US, annual direct costs related to HF are projected to exceed $70 billion by 2030.^2^ One of the most common causes of HF is acute myocardial infarction (AMI). Recent data suggest that ∼20% of patients who survive ST-segment elevation myocardial infarction (STEMI) develop HF, although this figure increases to roughly 30% among survivors of acute anterior STEMI even after prompt reperfusion therapy.^3^^,^^4^ As such, there is substantial interest in therapies to limit myonecrosis, particularly among patients with anterior STEMI.

To date, the only therapy that has been shown to reduce infarct size (IS) as an adjunct to primary percutaneous coronary intervention (PPCI) is supersaturated oxygen (SSO_2_) (ZOLL Medical).^5^^,^^6^ The Acute Myocardial Infarction With Hyperoxemic Therapy-II (AMIHOT-II) trial compared a 90-minute SSO_2_ infusion with no SSO_2_ among patients with a large anterior STEMI treated within 6 hours of symptom onset with PPCI. A prespecified Bayesian analysis combining AMIHOT-II data with similar patients from the AMIHOT-I trial demonstrated a 24% relative reduction (RR) in IS and noninferior 30-day rates of major cardiovascular (CV) events with the addition of SSO_2_ to PPCI, leading to FDA approval of SSO_2_ for this population.

Despite regulatory approval, there is no evidence of long-term clinical benefit with this therapy, as the available clinical trials were only powered for surrogate end points. Thus, there is considerable uncertainty as to the long-term cost-effectiveness of SSO_2_ therapy. To address this gap in knowledge, we used data from the AMIHOT-I and AMIHOT-II randomized trials, along with population data on long-term outcomes and costs after acute anterior STEMI, to develop a disease simulation model to project survival, QOL, and long-term costs after acute anterior STEMI.

## Materials and methods

**Figure 1:**
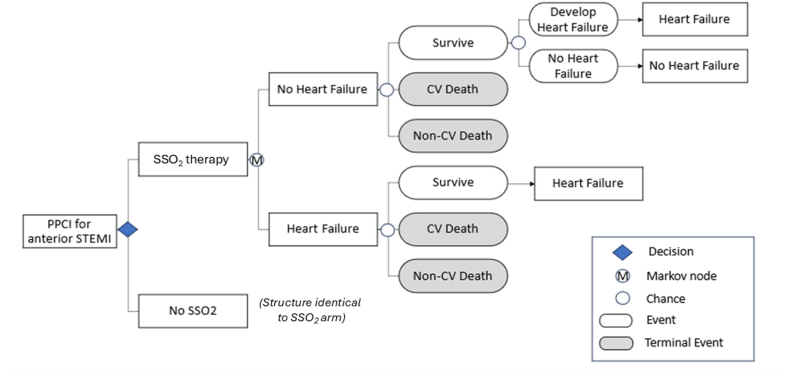
**Model structure.** Patients with anterior ST-segment elevation myocardial infarction (STEMI) undergoing primary percutaneous coronary intervention (PPCI) can be treated with supersaturated oxygen (SSO_2_) or no SSO_2_. After hospital discharge, all patients start in the no heart failure state. During each monthly cycle, patients are at risk for cardiovascular death (CV death) or noncardiovascular death (non-CV death), and surviving patients are at risk for developing heart failure. See Methods and Supplemental Material for details regarding key assumptions, model parameters, and data sources.

We developed a state-transition (Markov) model to project long-term outcomes after acute anterior STEMI treated with either SSO_2_ or no SSO_2_. The model was developed using TreeAge software (TreeAge Pro for Healthcare, v2025) and was structured as a decision tree with identical branches for the 2 treatment arms (Figure 1). Patients with acute anterior STEMI undergoing PPCI could be treated with SSO_2_ or no SSO_2_, and were initially in the no HF state. During each 1-month cycle after hospital discharge, patients could die from non-CV causes (non-CV death), die from CV causes (CV death), or survive. In addition, surviving patients could develop HF; once patients developed HF, it was assumed to persist indefinitely (HF state). Each cycle was associated with health state–specific health care costs and utilities, and associated risks for non-CV and cardiovascular mortality (CVM). Primary model outcomes were discounted costs and discounted quality-adjusted life-years (QALYs) over a lifetime horizon. Secondary outcomes included undiscounted QALYs and discounted and undiscounted life-years.

### Model inputs and sources

Characteristics of the model cohort mirrored those of the AMIHOT-II trial population (mean age 61 years; 78% men; IS 25% of left ventricular myocardium).^5^ Other than the effects of SSO_2_ therapy (which were derived from the AMIHOT-II randomized trial), model assumptions were derived from population-based data sets wherever possible. When modeling assumptions were necessary, these assumptions were chosen to be conservative with respect to the benefit of SSO_2_ therapy.

### Transition probabilities

Model assumptions regarding patient characteristics, outcome probabilities (as a function of IS), and the effect of SSO_2_ on IS are summarized in Supplemental Table S1. The risk of developing HF during the first year after anterior STEMI was assumed to be a function of IS based on pooled data from 7 clinical trials (n = 1774 patients) published in 2024 by de Waha et al.^7^ This risk function was then calibrated to the absolute population risk derived from an analysis of the 1-year incidence of HF hospitalization among patients who underwent PPCI for anterior STEMI in 2024 and were included in the Nationwide Readmissions Database (n = 21,426), assuming that mean IS for the Nationwide Readmissions Database cohort was the same as in the control group of the AMIHOT-II trial.^8^ We used an analogous approach to model CVM as a function of IS, with background rates derived from contemporary Danish data on 10-year survival after STEMI and the relationship between IS and 1-year mortality after PPCI for anterior STEMI from de Waha et al.^7^^,^^9^ Because there are no published data on the relationship between IS and either incident HF or mortality beyond 1-year of follow-up, we made the conservative assumption that IS did not directly affect future risk of HF or excess CVM (except through its impact on prevalent HF) beyond the first year of follow-up in our model. The excess risk of CVM among patients with HF was derived from analysis of long-term survival among Medicare patients with anterior STEMI and was assumed to persist indefinitely (see Supplemental Appendix S1 for details).

In order to ensure that our model was calibrated to US health statistics, we estimated age-specific contributions to CV and non-CV mortality from US age and sex-based life tables, applying cause-of-death categories from the National Vital Statistics Report for 2019 to avoid distortions from the COVID-19 pandemic (see Supplemental Appendix S1).^10^^,^^11^ Age and sex-specific excess CVM rates were applied based on the age at which US life table–based mortality exceeded that of the Danish post-STEMI data (an age of 75 years for men and an age of 80 years for women). Age- and sex-specific non-CV mortality rates from US Vital Statistics were applied throughout the post-MI period.

The effect of SSO_2_ treatment on IS was based on the mean relative reduction (RR) seen in the AMIHOT-II trial, which was reestimated from the primary study data using Bayesian and Monte Carlo Markov Chain methods.^5^ Applying a posterior predictive/potential outcomes framework, we estimated a RR in IS of 23.8% (95% credible interval, 1.5% to 40.0%; see Supplemental Material for details).

### Costs

Costs were assessed from the perspective of the US health care system in 2023 US dollars and are summarized in Supplemental Table S2. Costs for background health care after anterior STEMI were estimated based on facility and physician payments from the 5% Medicare claims sample. Monthly background costs for the first 2 years after the index STEMI were assessed among patients without HF and applied to all patients for the first 2 years of the model; year 2 costs were applied to year 3 and thereafter. Incremental monthly costs for patients who developed HF were estimated from the Medicare dataset using multiple linear regression adjusting for age, sex, and comorbidities. SSO_2_ therapy costs ($10,528) were provided by Zoll Medical and included the acquisition cost for disposables ($7253), physician fee for administration, additional cardiac catheterization laboratory time associated with administration, nonphysician personnel, and capital equipment (which was amortized over a 5-year period).

### Utility weights

Utility weights associated with health states are summarized in Supplemental Table S3. Patient-level data from a national registry of AMI patients (Translational Research Investigating Underlying disparities in acute Myocardial infarction Patients’ Health status^12^), including 1-year health state and mortality outcomes, were used to estimate utilities from EQ-5D scores at baseline and at months 1, 6, and 12. Regression models were used to estimate utility weights for patients with and without HF, using multiple imputation of missing data to reduce bias. These utility weights were further adjusted for age, based on published data.^13^

### Cost-effectiveness analyses

The base case analysis assessed lifetime cost, life expectancy, and quality-adjusted life expectancy for patients treated with SSO_2_ therapy and standard care (no SSO_2_) over a lifetime horizon. Consistent with current US guidelines, all future costs and health benefits were discounted at 3%/y.^14^^,^^15^ The primary end point was the incremental cost-effectiveness ratio (ICER) for SSO_2_ compared with standard care in terms of cost per quality-adjusted year of life gained. Outcomes were also estimated within predefined subgroups according to sex and age categories (male and female, overall and at ages 50, 60, 70, and 80 years).

The impact of model parameters and assumptions on the cost-effectiveness of SSO_2_ was assessed using 1-way deterministic sensitivity analyses (for all parameters) and 2-way sensitivity analyses (for selected parameters). Overall model uncertainty was assessed using probabilistic sensitivity analysis (PSA) in which all model parameters were sampled from their associated distributions (2000 replicates). All model input values, distributions for PSA, ranges for deterministic sensitivity analyses, and sources are summarized in Supplemental Tables S1-S3. Threshold analyses were performed to determine the values for high-leverage model parameters that would lead to an ICER of <$120,000/QALY gained (good economic value), as recommended by 2025 ACC/AHA guidance.^16^

### Ethical statement

This study is based on the analysis of previously collected and publicly available aggregate data, and therefore, no IRB approval was required.

## Results

**Figure 2:**
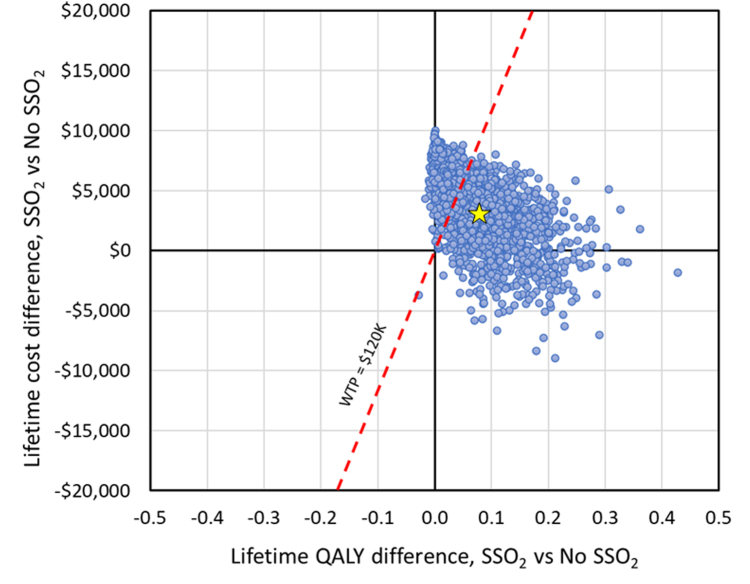
**Joint distribution of lifetime incremental cost and quality-adjusted life-years (QALYs) with supersaturated oxygen (SSO_2_) vs no SSO_2_.** Incremental lifetime costs and benefits with SSO_2_ vs no SSO_2_ are plotted on the cost-effectiveness plane with benefits expressed as QALYs. The yellow star represents base case estimates; the surrounding blue circles represent individual results for 2000 replicates of the study using probabilistic sensitivity analysis in which all model parameters were randomly sampled from their distributions. The dotted line represents a willingness-to-pay (WTP) threshold of $120,000 per QALY gained. The base case results demonstrated a gain of 0.083 QALYs at an incremental cost of $3205 per patient (after discounting), resulting in an incremental cost-effectiveness ratio of $38,415 per QALY gained.

**Figure 3:**
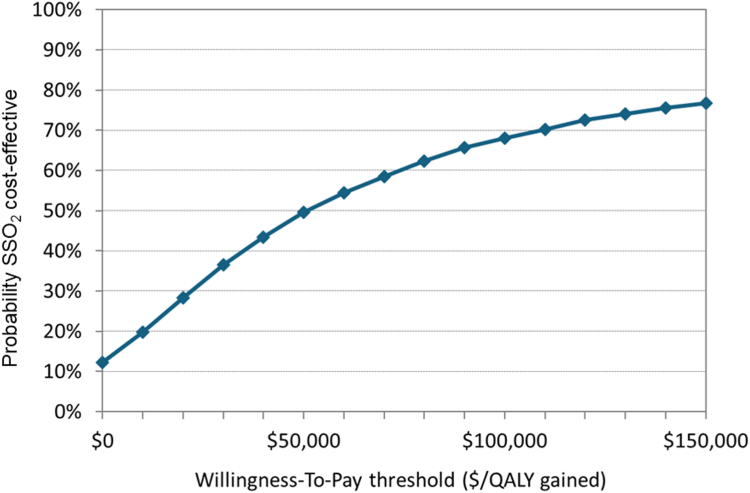
**Cost-effectiveness acceptability curve for supersaturated oxygen (SSO_2_) vs no SSO_2_.** This graph displays the probability that SSO_2_ is cost effective at a given willingness-to-pay threshold based on probabilistic sensitivity analysis. The probability that SSO_2_ is cost effective at a threshold of $120,000/QALY gained is 72.9%. QALY, quality-adjusted life-year.

In the base case analysis, our model projected that for patients similar to the AMIHOT-II study population, treatment with SSO_2_ compared with standard care would lead to a reduction in the rate of death or HF hospitalization at 1 year follow-up from 17.2% to 14.0%—driven largely by a reduction in new onset HF (from 16.8% to 13.3%). When these benefits were projected over a lifetime horizon, they led to a $7323/patient reduction in follow-up costs ($140,020 vs $147,343), driven by reduced costs of HF hospitalization and associated care. After incorporating the cost of SSO_2_ therapy ($10,528), discounted lifetime medical care costs were projected to be $3205/patient higher with SSO_2_ therapy compared with standard care ($150,548 vs $147,343). In addition to these economic benefits, quality-adjusted life expectancy was projected to increase by 0.083 QALYs with SSO_2_ vs standard care (10.776 vs 10.693 QALY, respectively), resulting in a base case ICER of $38,415 per QALY gained (Central Illustration). PSA demonstrated that SSO_2_ therapy was economically dominant (lower cost, greater quality-adjusted life expectancy) in 11% of model replicates. In addition, SSO_2_ therapy was projected to result in an ICER <$120,000/QALY gained (good economic value) in 73% of replicates (Figures 2 and 3).

**Central Illustration:**
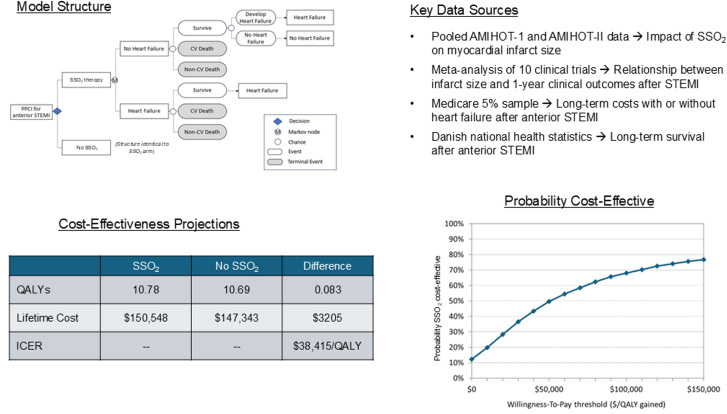
**Overview of study design (upper right-hand panel), key data sources (upper right-hand panel), and main results (lower left- and right-hand panels).** AMIHOT-I, Acute Myocardial Infarction With Hyperoxemic Therapy-I; AMIHOT-II, Acute Myocardial Infarction With Hyperoxemic Therapy-II; CV, cardiovascular; ICER, incremental cost-effectiveness ratio; PPCI, primary percutaneous coronary intervention; QALY, quality-adjusted life-year; SSO_2_, supersaturated oxygen; STEMI, ST-segment elevation myocardial infarction.

Key subgroup and sensitivity analyses are summarized in Table 1. The ICER for SSO_2_ was $40,605/QALY for men and $31,011/QALY for women. Among men, the ICER for SSO_2_ ranged from $19,976/QALY gained at an age of 50 years to $387,478/QALY gained at an age of 80 years, driven mainly by decreasing life expectancy and QALY gains with increasing age. Among women, the ICER for SSO_2_ ranged from $16,379/QALY gained at an age of 50 years to $236,119/QALY gained at an age of 80 years. The cost-effectiveness of SSO_2_ therapy was also highly sensitive to alternative assumptions regarding the duration of the effect of SSO_2_ on clinical outcomes. If we assumed that the effect of SSO_2_ on both CV mortality and new onset HF continued through 5 years (rather than 1 year in our base case), SSO_2_ therapy was projected to be an economically dominant strategy, increasing quality-adjusted life expectancy by 0.22 QALYs (compared with 0.08 QALYs in our base case) and reducing lifetime health care costs by >$7000/patient.

**Table 1:** Lifetime cost, effectiveness, and cost-effectiveness of SSO_2_ for base case and selected sensitivity and subgroup analyses

	Lifetime discounted costs			Lifetime discounted QALY			ICER ($/QALY)	% SSO_2_ dominant	% ICER <$120,000 per QALY gained
	SSO_2_	Standard care	Δ	SSO_2_	Standard care	Δ			
Base case	$150,548	$147,343	$3205	10.78	10.69	0.083	$38,415	11%	73%
Sex									
Male	$148,383	$145,094	$3289	10.6	10.52	0.081	$40,605	11%	71%
Female	$159,085	$156,201	$2884	11.47	11.37	0.093	$31,011	14%	77%
Age (male), y									
50	$176,345	$174,023	$2322	12.97	12.86	0.116	$19,976	20%	94%
60	$151,327	$148,146	$3181	10.85	10.76	0.085	$37,567	13%	85%
70	$118,268	$113,801	$4467	8.13	8.08	0.05	$89,018	2%	59%
80	$77,393	$71,130	$6263	4.88	4.86	0.016	$387,478	0%	5%
Age (female), y									
50	$184,205	$182,152	$2053	13.61	13.49	0.125	$16,379	25%	94%
60	$161,904	$159,120	$2784	11.7	11.61	0.096	$29,137	15%	90%
70	$129,473	$125,451	$4022	9.03	8.97	0.061	$66,191	4%	69%
80	$88,235	$82,459	$5776	5.72	5.7	0.024	$236,119	0%	17%
Duration of effects of IS on HF and CV mortality									
5 y	$228,900	$236,265	($7365)	10.18	9.95	0.224	SSO_2_ dominant	81%	90%
5 y (tapered)^a^	$234,389	$235,174	($786)	10.11	9.96	0.15	SSO_2_ dominant	49%	94%

Values in parentheses represent negative values.

CV, cardiovascular; HF, heart failure; ICER, incremental cost-effectiveness ratio; IS, infarct size; QALY, quality-adjusted life-year; SSO_2_, supersaturated oxygen.

a Effect of IS on HF and CV mortality tapered to no effect at 5 years.

**Figure 4:**
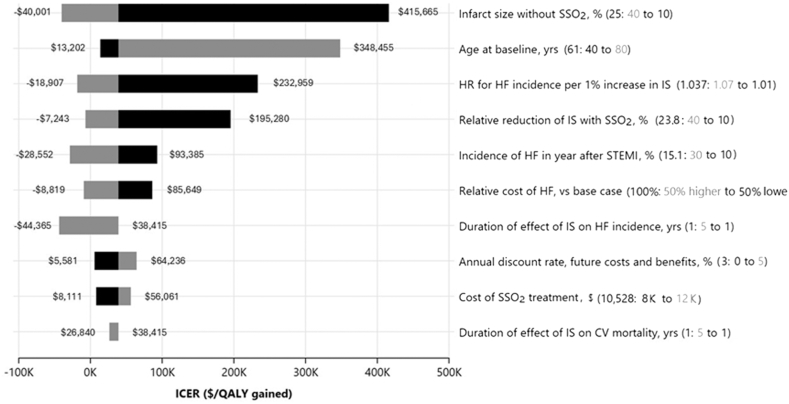
**Tornado diagram.** The figure shows the impact of variations in each individual model input on the incremental cost-effectiveness ratio (ICER) for supersaturated oxygen (SSO_2_) vs no SSO_2_. Gray bars correspond to gray values for each model input, which are listed in parentheses next to each parameter. CV, cardiovascular; HF, heart failure; IS, infarct size; STEMI, ST-segment elevation myocardial infarction.

**Figure 5:**
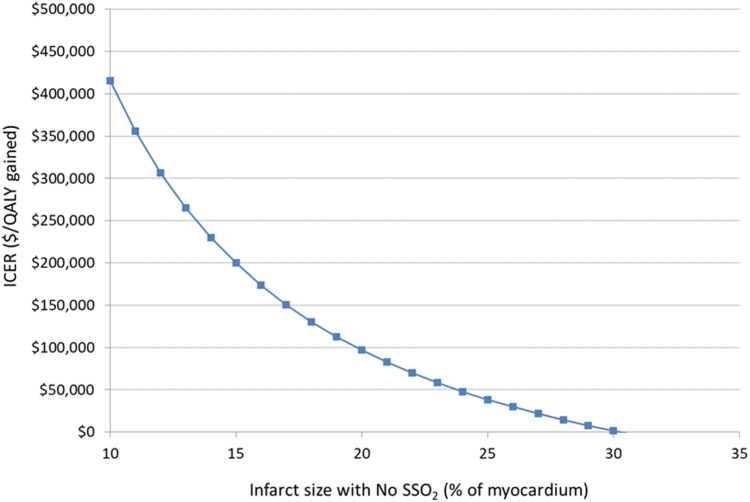
**One-way sensitivity analysis: Incremental cost-effectiveness ratio (ICER) vs infarct size with no supersaturated oxygen (SSO_2_).** This graph displays the ICER for SSO_2_ vs no SSO_2_ over a range of values of infarct size without SSO_2_ therapy. At an infarct size of 20%, the ICER is $96,830 per quality-adjusted life-year (QALY) gained. At an infarct size of 15%, the ICER is $199,777 per QALY gained relative to no SSO_2_.

**Figure 6:**
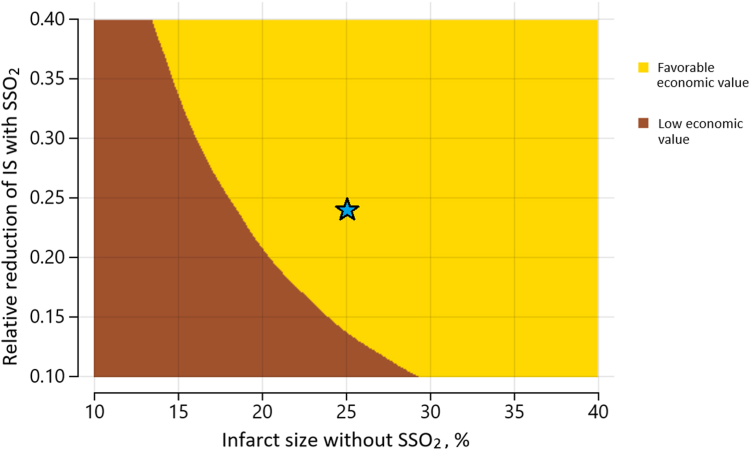
**Two-way sensitivity analysis.** This figure shows the relationship between variation in infarct size without supersaturated oxygen (SSO_2_) and the relative reduction in infarct size with SSO_2_ therapy on the incremental cost-effectiveness ratio (ICER) for SSO_2_. The yellow shaded area represents the combinations of these variables for which SSO_2_ provides acceptable economic value (ICER <$120,000/QALY), while the dark red shaded area represents combinations for which SSO_2_ provides low economic value (ICER ≥$120,000/QALY). The star represents the base case scenario. QALY, quality-adjusted life-year.

Deterministic 1-way sensitivity analyses were performed by varying each model variable over its plausible range; results are summarized as a tornado diagram of the 10 most influential variables and their impact on the projected ICER for SSO_2_ (Figure 4). The IS in the standard care group had the greatest impact on the cost-effectiveness of SSO_2_. If the IS with standard care was 10% (rather than 25% in the base case), the ICER for SSO_2_ therapy was projected to increase to $415,665/QALY (Figure 5). On the other hand, if the IS with standard care was 30%, SSO_2_ was projected to be highly cost effective, with an ICER of $1793/QALY gained, and if IS was 40% (a value that may be seen in large anterior STEMI because of proximal LAD occlusion), SSO_2_ was projected to be an economically dominant strategy with lifetime cost savings of $8448/patient. The only other model variables with a major impact on the cost-effectiveness of SSO_2_ therapy were the effect of IS on HF incidence and the effect of SSO_2_ on IS. All other variables had at most a modest impact on the cost-effectiveness of SSO_2_ as indicated by a maximum ICER <$100,000/QALY gained. Interestingly, over a plausible range, the cost of SSO_2_ therapy had only a minimal impact on the cost-effectiveness of SSO_2_. Threshold analyses demonstrating the values of key parameters associated with good economic value are summarized in Table 2. SSO_2_ therapy was projected to be cost effective (ICER <$120,000/QALY) as long as the IS with standard care was at least 18.6% (with all other factors unchanged from baseline) or the RRR in IS with SSO_2_ was at least 13.9%. The ICER for SSO_2_ remained <$120,000/QALY for men aged 73 years or less and for women aged 75 years or less. Two-way sensitivity analysis examining the impact of simultaneous variation of both IS with standard care and the RR in IS with SSO_2_ is summarized in Figure 6.

**Table 2:** Threshold analyses for key parameters

Model parameter	Base case value	Threshold for ICER < $120,000/QALY gained
Age (male), y	61	73
Age (female), y	61	75
Infarct size with standard care (no SSO_2_), %	25.0	18.6
Relative reduction of infarct size with SSO_2_, %	23.8	13.9
Incremental cost of SSO_2_ therapy (including procedural costs and physician costs)	$10,528	$17,334

ICER, incremental cost-effectiveness ratio; QALY, quality-adjusted life-year; SSO_2_, supersaturated oxygen.

## Discussion

Despite substantial advances in stent technology, antithrombotic therapy, and procedural techniques for PPCI over the past 3 decades, none of these advances has impacted myocardial IS—one of the major drivers of adverse long-term outcomes after AMI, including new onset HF and late mortality. Indeed, the only therapy proven to reduce IS after AMI is intracoronary SSO_2_.^5^ Despite FDA approval in 2019, however, many critical questions regarding this therapy remain unanswered, including its impact on long-term mortality and costs.

To address these important gaps in knowledge, we developed a disease simulation model to project long-term outcomes (including HF and mortality), costs, and cost-effectiveness of adjunctive SSO_2_ therapy for patients undergoing PPCI for acute anterior STEMI. Key model assumptions were based on the published literature and publicly available data including the following: (1) the effect of SSO_2_ on IS, (2) the relationship between IS and 1-year clinical outcomes after anterior STEMI, and (3) contemporary population-based estimates of long-term mortality after anterior STEMI. In addition, we used population-based data from US patients to estimate the relationship between new onset HF after anterior STEMI and both long-term mortality and health care related costs. Finally, we used data from a 5000-patient prospective AMI registry to estimate the relationship between HF after AMI and QOL. These data allowed us to project long-term survival, life expectancy, quality-adjusted life expectancy, and total health care costs for a cohort of patients with anterior STEMI undergoing PPCI who were treated with SSO_2_ therapy or standard care (no SSO_2_).

Based on these data inputs, our model projected that treatment with SSO_2_ would increase discounted quality-adjusted life expectancy from 10.693 QALYs to 10.776 QALYs—a gain of 0.083 QALYs, which was driven by both reduced mortality and improved QOL from avoidance of HF. Although initial treatment costs were ∼$10,500/patient higher with SSO_2_ therapy (reflecting the cost of the treatment and the additional procedural time), our model projected that follow-up costs would be reduced by more than $7000/patient, resulting in a net lifetime cost of ∼$3200/patient and an ICER of $38,415/QALY gained. Although model projections were sensitive to several parameters including IS without SSO_2_, the RR of IS with SSO_2_, and the relationship between IS and subsequent mortality and incident HF, PSA (in which all model parameters were varied over their plausible ranges) demonstrated a 73% probability that SSO_2_ is cost effective at a willingness-to-pay threshold of <$120,000/QALY gained from the perspective of the US health care system. Treatment of patients aged 60 years or less (who are projected to derive greater life expectancy gains as well as greater cost offsets) was projected to be cost effective in at least 85% of trial replicates.

### Comparison with other therapies

To place our findings in context, it is useful to compare the cost-effectiveness of SSO_2_ with that of other therapies for the treatment of HF. In an economic analysis of the Cardiovascular Outcomes Assessment of the MitraClip Percutaneous Therapy for Heart Failure Patients With Functional Mitral Regurgitation (COAPT) trial, Baron et al^17^ projected that for patients with HF and severe secondary mitral regurgitation, transcatheter mitral edge-to-edge repair (M-TEER) would increase quality-adjusted life expectancy by 0.82 QALYs at an incremental lifetime cost of $45,648, resulting in an ICER of $55, 600/QALY gained. A model-based cost-effectiveness analysis of sacubitril-valsartan for patients with HF with reduced ejection fraction (based on results of the PARADIGM-HF trial) projected an increase in quality-adjusted life expectancy of 0.75 QALYs at an incremental cost of $38,633, yielding an ICER of $50,959/QALY gained.^18^ Finally, a recent model-based cost-effectiveness analysis projected that for patients with HF and preserved ejection fraction, dapagliflozin would increase both quality-adjusted life expectancy and costs (by 0.53 QALY and $45,509, respectively), yielding an ICER of $85,554/QALY gained.^19^ Thus, it appears that the cost-effectiveness of SSO_2_ for a typical patient undergoing PPCI for anterior STEMI compares favorably with many other accepted therapies for treatment of HF.

The greater projected gains in quality-adjusted life expectancy with M-TEER, sacubitril-valsartan, and dapagliflozin relative to that with SSO_2_ are not surprising, because all 3 of these therapies were studied in patients with HF, whereas SSO_2_ was studied in patients with AMI, only a fraction of whom will go on to develop HF. Nonetheless, as a single-time treatment with a relatively modest up-front cost, the lifetime incremental cost of SSO_2_ was much lower than that of either M-TEER (with a treatment cost of >$50,000) or with the 2 drugs, although it is likely that the cost-effectiveness of sacubitril-valsartan will improve substantially with recent approval of several generic formulations in the US.

### Modeling considerations

It is important to acknowledge that our findings are based on computer simulation modeling rather than on empirical data on long-term outcomes and costs from a randomized clinical trial. This approach was necessary because the clinical trials leading to FDA approval of SSO_2_ therapy were powered for the surrogate end point of IS reduction rather than for clinical events. Although it would be ideal to conduct an RCT powered to demonstrate clinical benefit, it is likely that the sample size for such a trial would be prohibitive. Based on the projected 1-year rates of death or HF hospitalization from our model, a sample size of >3000 patients would be necessary to demonstrate a reduction in the 1-year incidence of death or HF hospitalization from 17% to 13% (as suggested by our model). To date, the only randomized device trials of this size in patients with STEMI have been postapproval studies of aspiration thrombectomy.^20^^,^^21^

In the absence of empirical outcomes data, the validity of the model projections thus rests on the validity of its assumptions, which were derived from a combination of high quality randomized trial data (for treatment efficacy), pooled data from randomized trials (for the relationship between IS and clinical outcomes), and population data (for model calibration and for defining the relationship between new onset HF and long-term survival and costs). Importantly, the causal association between IS and clinical outcomes after AMI (especially mortality), which is central to our model, was based on anterior STEMI-specific pooled data from 7 clinical trials and has also been seen in several independent studies.^7^^,^^22^^,^^23^ Although a 2017 meta-regression analysis of the relationship between IS reduction and mortality benefit of various therapies did not demonstrate a statistically significant correlation,^24^ given the neutral effects of virtually all of the therapies on IS, the study was markedly underpowered to detect such an effect. It is nonetheless reassuring that the point estimate for the relationship between IS and mortality from that study was virtually identical to our base case assumption.

Moreover, where assumptions beyond the available data were necessary, our guiding principle was to choose the assumption that biased the results against SSO_2_. For example, the only data available regarding the relationship between IS and clinical outcomes were for 1-year outcomes. We, therefore, made the conservative assumption that this effect would be limited to the first year after AMI. Under less conservative modeling assumptions (eg, benefit of SSO_2_ on mortality and HF persisting for 5 years), SSO_2_ therapy was projected to be an economically dominant strategy.

### Limitations

This study should be interpreted in light of several limitations. First, although we modeled a cohort with a mean age of 61 years, we used Medicare claims data to estimate post-STEMI health care costs. Because Medicare payments are generally lower than those for private insurance, it is likely that our estimates of the cost offsets associated with avoidance of HF are underestimated, which would bias our analysis against SSO_2_. Second, although our analysis captured most health care costs (including inpatient, outpatient, home health, rehabilitation, skilled nursing facility, and physician costs), medication costs were not available in the Medicare standard analytic files. Because HF was more frequent in the standard care arm, exclusion of these costs almost certainly biased the analysis against SSO_2_ as well. Third, given the sensitivity of our analysis to modest reduction in IS with standard care, it is possible that future advances in STEMI management may warrant reconsideration of our findings using updated model parameters. Finally, our analysis was conducted from the perspective of the US health care system. Therefore, our findings do not apply to other countries that may have differences in both outcomes and resource costs. In addition, because our analysis takes a long-term perspective, it does not relate directly to hospital finances, which currently depend on the balance between cost and reimbursement for an episode of care.

## Conclusions

Based on our disease simulation model with conservative assumptions derived from randomized trials and population-based outcomes data, SSO_2_ therapy for patients with acute anterior STEMI is projected to be a cost effective adjunct to PPCI from the perspective of the US health care system.

## Footnotes

To access the supplementary material accompanying this article, visit the online version of the *Journal of the Society for Cardiovascular Angiography & Interventions* at 10.1016/j.jscai.2026.105321.
