# Supplementary material for: Cost-Effectiveness of Supersaturated Oxygen Delivery for Infarct Size Reduction in Patients With Anterior ST-Segment Elevation Myocardial Infarction
Source: J Soc Cardiovasc Angiogr Interv. 2026 May 7;5(7):105321. doi: 10.1016/j.jscai.2026.105321 (PMC13400098; doi:10.1016/j.jscai.2026.105321)
Supplement: Supplemental Material [file mmc1.docx]

**Supplemental Appendix S1**

**Cost-Effectiveness of Supersaturated Oxygen Delivery
for Infarct Size Reduction in Patients with Anterior STEMI**

**Detailed Methods for Estimation of Relative Reduction in Infarct Size
with SSO_2_**

The previously published analysis of the AMIHOT-1 anterior STEMI subgroup and the AMIHOT-2 confirmatory trial used a Bayesian hierarchical model for a shifted log transformed infarct size (Y=log[IS+10])[1]. However, parameters from this model are not directly interpretable as log relative reduction (RR) in infarct size. In order to obtain an effect estimate and 95% credible interval for the RR consistent with the published analysis, we used a potential outcomes approach. For each of the simulated parameter vectors obtained in the Markov Chain Monte Carlo (MCMC) output for the original analysis, we simulated a large number of potential outcome pairs differing only by the causal effect of the treatment. For each of these pairs we then back-transformed to the IS scale via the inverse of the shifted log transformation. Finally, for each pair we calculated the RR and then averaged across the simulated potential outcome pairs. Repeating this across all of the MCMC simulations yielded a posterior distribution of RR in infarct size, from which we derived the posterior median as the estimate with associated 95% equal-tailed credible interval as the parameter estimate.

**Supplemental Table S1.** Model Assumptions and Data Sources - Clinical Outcomes

| **Model Parameter** | **Distribution** | **Base Case**  **Value** | **Variability Parameter*** | **Source(s)/Reference Number(s)** |
| --- | --- | --- | --- | --- |
| *Cohort Characteristics* | | | | |
| Age, years | Normal | 61 | 12.1 | [1] |
| Male, % | Beta | 77.8 | 10 | [1] |
| Infarct size, % | Beta | 25 | 2.3 | [1] |
| Annual incidence of HF after anterior STEMI treated with primary PCI, % | N/A | 15.1 | 10-30 (range) | Primary analysis of 2024 Nationwide Readmissions Database for patients with acute anterior STEMI undergoing primary PCI (see Methods for details) |
| Annual rate of CV mortality among patients with anterior STEMI treated with PCI, % | N/A | 0.95 | 0.6-2 (range) | [2] |
| *Effect of SSO_2_ therapy* | | | | |
| Relative reduction of IS with SSO_2_, % | Beta | 23.8 | 7.2 | Pooled analysis of AMIHOT-I anterior MI patients treated w/in 6 hours and AMIHOT-II |
| *Effects of IS on clinical outcomes* | | | | |
| HR for new HF (per 1% increase in IS), % | Normal | 1.037 | 0.001 | [3] |
| HR for CV mortality (per 1% increase in IS) | Normal | 1.035 | 0.001 | [3] (based on HR for all-cause mortality) |
| Duration of effect of IS on outcomes, years | N/A | 1 | N/A | Conservative assumption. Varied between 1 and 5 years in sensitivity analyses. |
| *Effect of HF on mortality* | | | | |
| Relative Risk of CV mortality with vs without HF | LogNormal | 2.48 | 2.36 | Primary analysis of 2017-2019 5% CMS Standard Analytic Files claims data for patients with anterior STEMI |

*Variability parameter used to estimate distributional parameters for probabilistic sensitivity analysis

For beta, gamma, and Normal distributions: Variability parameter = standard deviation
For lognormal distribution: Variability parameter = median

CV = cardiovascular; IS = infarct size

**Supplemental Table S2.** Model Assumptions and Data Sources - Utilities

| Model Parameter |  | Mean (beta distribution) | | | 95% CI | Source(s)/Reference Number(s) | |
| --- | --- | --- | --- | --- | --- | --- | --- |
| *Health State Utilities* | | | | | | | |
| No heart failure | | | | | | | |
| Baseline |  | | 0.781 | 0.555 – 0.833 | | | Primary analysis of TRIUMPH registry [4]* |
| At 1-month follow-up |  | | 0.827 | 0.662 – 0.992 | | | “ |
| At 6-month follow-up |  | | 0.828 | 0.662 – 0.994 | | | ” |
| At 12-month follow-up |  | | 0.818 | 0.654 – 0.982 | | | ” |
| Relative Reduction of utility with HF | | | | | | | |
| Baseline |  | | 0.889 | 0.833 – 0.944 | | | Primary analysis of TRIUMPH registry [4] |
| At 1-month follow-up |  | | 0.874 | 0.811 – 0.937 | | | ” |
| At 6-month follow-up |  | | 0.859 | 0.895 – 0.965 | | | ” |
| At 12-month follow-up |  | | 0.868 | 0.802 – 0.934 | | | ” |
| *Disutilities* | | | | | | | |
| Disutility: first HF hospitalization |  | | 0.10 | 0.05 – 0.15 | | [5, 6] | |
| Disutility per year of age |  | | 0.0006 | 0 - 0.001 | | [7] | |

HF = heart failure

*See Methods Section (Utility Weights sub-section) for details of analysis relating utility weights to prevalent heart failure from the TRIUMPH Registry

**Supplemental Table S3.** Model Assumptions and Data Sources – Costs

| Model Parameter | Mean  (gamma distribution) | Standard  deviation | Source(s)/Reference Number(s) |
| --- | --- | --- | --- |
| Monthly post-STEMI | $637 | $90 | Primary analysis of 2017-2019 5% CMS Standard Analytic Files claims data for patients with anterior STEMI |
| Monthly increment  with HF, year 1 | $3472 | $200 | “ |
| Monthly increment  with HF, after year 1 | $1633 | $120 | “ |
| SSO_2_ therapy (inclusive) | $10,528 | N/A | Resource accounting based on expert opinion |
| Disposable supplies | $7515 | N/A | Current list pricing (Zoll Medical) |
| Physician fee for  SSO2 infusion | $668 | N/A | Medicare fee schedule for acute MI PCI [8] |
| Cath Lab Overhead and Depreciation | $1509 | N/A | Cath lab cost per minute based on hospital accounting data [9, 10] |
| Non-physician personnel | $101 | N/A | Personnel cost per minute based on hospital accounting data |
| SSO2 console | $735 | N/A | List price of $75,000 amortized over 5 years (21 procedures/year) |

HF = heart failure

**REFERENCES**

1. Stone, G.W., et al., Effect of supersaturated oxygen delivery on infarct size after percutaneous coronary intervention in acute myocardial infarction. Circ Cardiovasc Interv, 2009. **2**(5): p. 366-75.

2. Thrane, P.G., et al., 10-Year Mortality After ST-Segment Elevation Myocardial Infarction Compared to the General Population. J Am Coll Cardiol, 2024. **83**(25): p. 2615-2625.

3. de Waha, S., et al., Relationship Between Infarct Artery, Myocardial Injury, and Outcomes After Primary Percutaneous Coronary Intervention in ST-Segment-Elevation Myocardial Infarction. J Am Heart Assoc, 2024. **13**(18): p. e034748.

4. Arnold, S.V., et al., Translational Research Investigating Underlying Disparities in Acute Myocardial Infarction Patients' Health Status (TRIUMPH): design and rationale of a prospective multicenter registry. Circ Cardiovasc Qual Outcomes, 2011. **4**(4): p. 467-76.

5. Lewis, E.F., et al., Impact of cardiovascular events on change in quality of life and utilities in patients after myocardial infarction: a VALIANT study (valsartan in acute myocardial infarction). JACC Heart Fail, 2014. **2**(2): p. 159-65.

6. Di Tanna, G.L., et al., Health State Utilities of Patients with Heart Failure: A Systematic Literature Review. Pharmacoeconomics, 2021. **39**(2): p. 211-229.

7. Magnuson, E.A., et al., Cost-Effectiveness of Percutaneous Coronary Intervention Versus Bypass Surgery for Patients With Left Main Disease: Results From the EXCEL Trial. Circ Cardiovasc Interv, 2022. **15**(7): p. e011981.

8. CMS, Revisions to Payment Policies under the Medicare Physician Fee Schedule Quality Payment Program and Other Revisions to Part B for CY 2022: CMS-1751-F_Direct PE Inputs. 2021: <https://www.cms.gov/medicaremedicare-fee-service-paymentphysicianfeeschedpfs-federal-regulation-notices/cms-1751-f>.

9. Ballard, D.H., et al., Medical 3D Printing Cost-Savings in Orthopedic and Maxillofacial Surgery: Cost Analysis of Operating Room Time Saved with 3D Printed Anatomic Models and Surgical Guides. Acad Radiol, 2020. **27**(8): p. 1103-1113.

10. Pashankar, D.S., et al., A Quality Improvement Project to Improve First Case On-time Starts in the Pediatric Operating Room. Pediatr Qual Saf, 2020. **5**(4): p. e305.
